# Unlocking the Potential of Extracellular Vesicles in Cardiovascular Disease

**DOI:** 10.1111/jcmm.70407

**Published:** 2025-02-05

**Authors:** Hanbin Li, Lu Wang, Hongxin Cheng, Qing Zhang, Shiqi Wang, Wen Zhong, Chengqi He, Quan Wei

**Affiliations:** ^1^ Rehabilitation Medicine Center and Institute of Rehabilitation Medicine, West China Hospital Sichuan University Chengdu China; ^2^ Key Laboratory of Rehabilitation Medicine in Sichuan Province, West China Hospital Sichuan University Chengdu China

**Keywords:** CVD, EVs, exosomes, microparticles

## Abstract

Extracellular vesicles (EVs) are micro‐nanoscale biological particles encapsulated by phospholipid bilayers, which regulate cell migration, angiogenesis and tumour cell growth by transmitting various biomolecules such as nucleic acids and proteins. EVs are composed of exosomes, microparticles and apoptotic bodies. Its benefits pass through biofilms and are not degraded by various enzymes, so it can be used as a biomarker in potential diseases and has attracted much attention from researchers. Current studies have found that EVs are involved in the development of various cardiovascular diseases (CVD), such as heart failure and myocardial ischemia–reperfusion injury. In addition, stem cell‐derived EVs play an important role in the diagnosis and treatment of a variety of CVD. In this review, we present the biological features of EVs, the role of EVs in various CVD, and the challenges they encounter in the treatment of CVD.

## 1 Introduction

Extracellular vesicles (EVs) are a diverse group of lipid bilayer membranous nanoscale particles derived from cells [1, 2, 3], of which exosomes have generally been considered as a means of mediating system information exchange and long‐distance interactions between cells. Cardiovascular diseases (CVD) are one of the leading causes of death worldwide [4], and intercellular communication and coordination between different types of cells are essential for the integrity and normal function of organs. Cell‐to‐cell communication in the heart is mediated through cell‐to‐cell contacts, cell‐matrix interactions, and extracellular biochemical signals. EVs, including exosomes, microvesicles (MVs) and apoptotic bodies [2, 3, 4], transfer functional cargos like nucleic acids, proteins, and lipids from source cells to target cells, which are pivotal in cardiovascular communication and disease modulation [5]. The biogenesis and size variations of EVs contribute to their functional specificity in pathophysiological processes in diseases like heart failure (HF), atherosclerosis (AS), myocardial infarction (MI) and more [6, 7].

Recent advancements in cardiovascular research have highlighted EVs derived from various cell types, such as cardiomyocytes, endothelial cells (ECs) and smooth muscle cells (SMCs) [2, 8]. These EVs play crucial roles in regulating the complex mechanisms underlying CVD. The cargos of EVs, encased within their protective lipid bilayers, are intricately linked with CVD pathologies, vary significantly in patients, and are detectable in body fluids like serum and urine, making EVs promising candidates for non‐invasive biomarkers in CVD diagnosis and prognosis [9]. Further, the therapeutic potential of EVs in CVD is being rigorously explored. Their advantages, including ease of access, potential for modification, stability for storage, low immunogenicity and capability to traverse biological barriers, position them as innovative means of CVD management.

**FIGURE 1:**
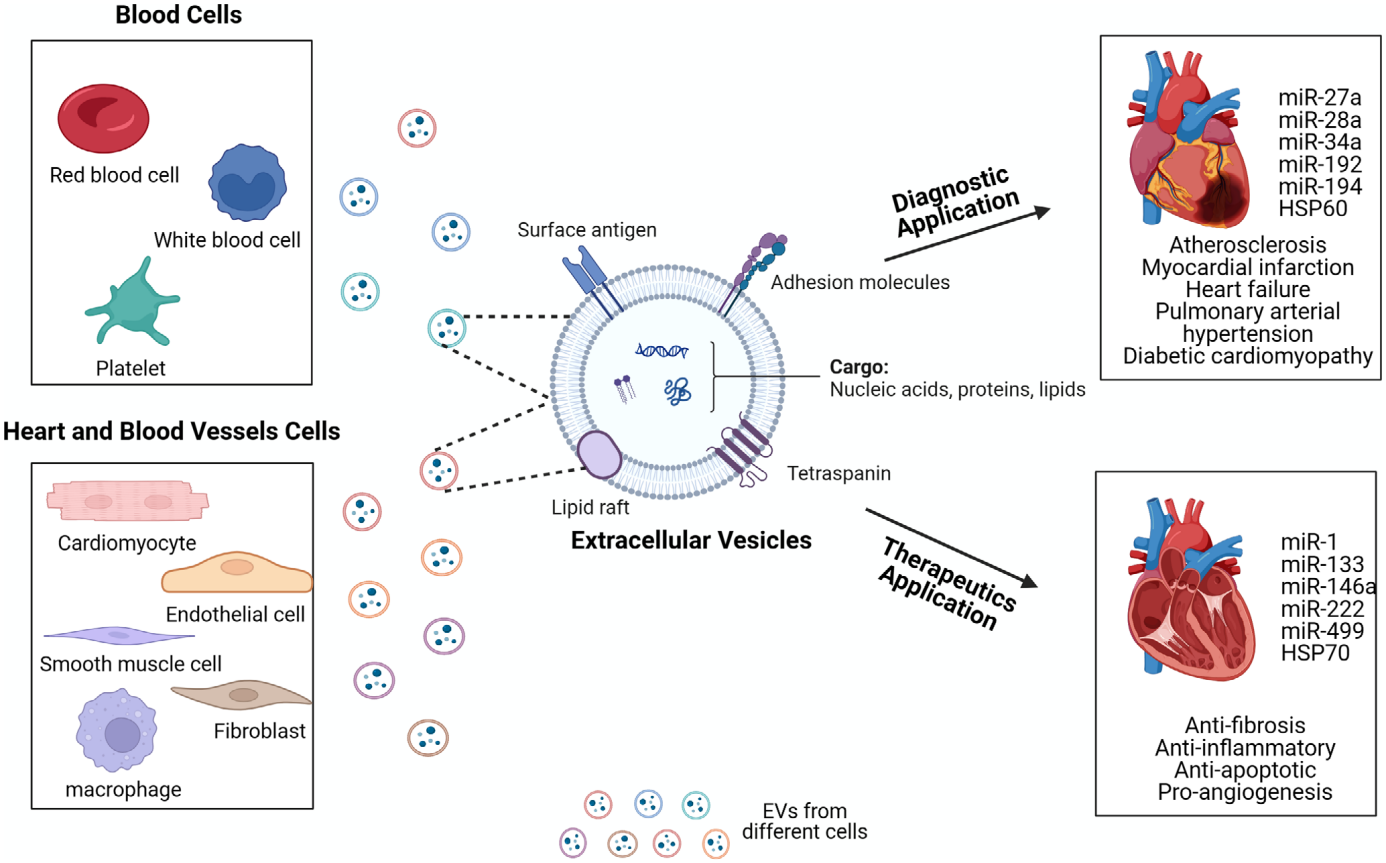
Origins, contents and roles of extracellular vesicles in cardiovascular diseases. Cardiovascular system‐related cells, including cardiomyocytes, endothelial cells, fibroblasts, platelets, smooth muscle cells, macrophages, red blood cells and white blood cells, can release extracellular vesicles (EVs). Under physiological conditions, EVs play vital roles in preserving normal cardiac structure and function. However, under pathological conditions, their composition changes, contributing to the progression of cardiovascular diseases (CVD). As a result, EVs offer significant potential for monitoring and treating CVD.

The primary aim of this review is to elucidate the roles of EVs in CVD, emphasising intercellular signalling function and therapeutic applications of EVs (Figure 1). Then, we will detail how EVs from cardiomyocytes, ECs, and macrophages modulate the pathophysiology of key CVD such as AS, MI, HF, and through the transfer of proteins, lipids and microRNAs (miRNAs). EVs' protein cargo includes various functional proteins, such as cytokines, growth factors, metalloproteinases and glycosidases, offering insights for diagnostics, therapeutic development, and predictive biomarkers in CVD. We also briefly introduce how miRNA‐containing EVs play a dual role in CVD by regulating gene expression in recipient cells, influencing cardiac remodelling and serving as therapeutic targets. Additionally, the potential of EVs as novel therapeutic delivery vehicles and predictive biomarkers will be explored, highlighting their implications for advancing cardiovascular therapy and diagnostics.

Lastly, we summarised the current limitations of EVs in clinical practice. While EVs show promising potential in CVD applications, several challenges hinder their translation. Critical gaps include the lack of standardised production protocols, storage difficulties, uncertainties regarding biodistribution and targeted delivery, and the need for high‐quality clinical trials to validate safety and efficacy. Overcoming these barriers is essential to bridge the gap between fundamental research and clinical use.

## 2 The Physiology of EVs in CVD

### 2.1 The Biogenesis and Release of EVs

Exosomes biogenesis is a precise intracellular event. First, exosomes formation begins with endocytosis of the cell membrane, where external substances enter the cell by endocytosis to form early‐sorting endosomes (ESEs), which can exchange substances with the trans‐Golgi network and endoplasmic reticulum [10]. As endosomes mature, they gradually transform into late‐sorting endosomes (LSEs), subsequently mature into MVBs, and form ILVs on the endosomal membrane. ILVs are direct precursors of exosomes. Thus the formation of ILVs is one of the core steps in exosomes biogenesis [11]. The formation of ILVs is dependent on the Endosomal Sorting Complex Required for Transport (ESCRT) [12] complexes (ESCRT‐0, ‐I, ‐II and ‐III) and their core proteins, such as ESCRT‐III, ALIX, and tumour susceptibility gene 101 (TSG101), which promote the generation of ILVs by regulating inward budding of membranes and separation of vesicles. Next, mature MVBs are transported to the cell membrane or lysosomes through the participation of molecules such as Ras‐associated binding (Rab) GTPases. When fused to lysosomes, ILVs are degraded, while when fused to membranes, endosomes are released into the extracellular space and become exosomes. In addition to the classical ESCRT‐mediated pathway, exosomes formation can also be achieved through ESCRT‐independent pathways such as lipids and Rab GTPases. For example [13], lipid raft‐rich regions of the cell membrane may directly bend inward during endocytosis to form ILVs. This pathway does not rely on ESCRT complexes but still requires the involvement of specific lipids and membrane proteins, such as tetraspanins (e.g. CD63, CD81, etc.), which usually play an important role in the formation and function of exosomes. Similarly, Rab GTPases [14] are involved in ESCRT‐mediated exosomes biogenesis, but they also regulate exosomes formation independently of the ESCRT pathway. Rab GTPases such as Rab27a, Rab35 and Rab11 are able to promote the release of exosomes by regulating the trafficking of MVBs as well as fusion with cell membranes. In the absence of ESCRT, these Rab proteins may directly contribute to the formation or release of endosomal vesicles by interacting with membrane proteins and other transport factors.

The biogenesis of microvesicles (MVs) appears to be formed by outward budding and fission of the plasma membrane, usually not involving the formation of endosomes and requiring concentrated molecular rearrangements in the plasma membrane, which include changes in lipid composition, proteins and Ca^2+^. For example, amino acid phospholipid transferases, scramblases and calpains drive asymmetric rearrangement of membrane phospholipids, resulting in physical bending of the membrane and remodelling of the actin cytoskeleton, favouring membrane budding and formation of MVs. Cytoskeletal elements and their regulators are also required for MVs generation. Increasing evidence suggests that formation of MVs is highly correlated with regulation of cytoskeletal elements by small GTPases [15], such as the Rho (RAS homology) family and ADP‐ribosylation factor (ARF), and in cancer cells, Rho‐associated coiled coils containing protein kinases and the GTP‐binding protein ARF6 are considered positive regulators of vesicle budding [16]. Part of the mechanism resembles the described extracellular budding of retroviral particles: targeting the site of MVs emergence through affinity for lipids or anchoring directly to the membrane substance of the plasma membrane.

Apoptotic bodies are also an EVs subtype produced by the budding of dead cells from the plasma membrane during apoptosis [17] and are usually recognised and phagocytosed by phagocytes [17]. It has been shown that apoptotic body formation is regulated by apoptotic cell breakdown, including ROCK, Pannexin‐1 and PlexinB2 [18]. Compared to exosomes and exosomes, studies on apoptotic bodies as an EVs subtype are limited.

### 2.2 The Cargos in CVD

**FIGURE 2:**
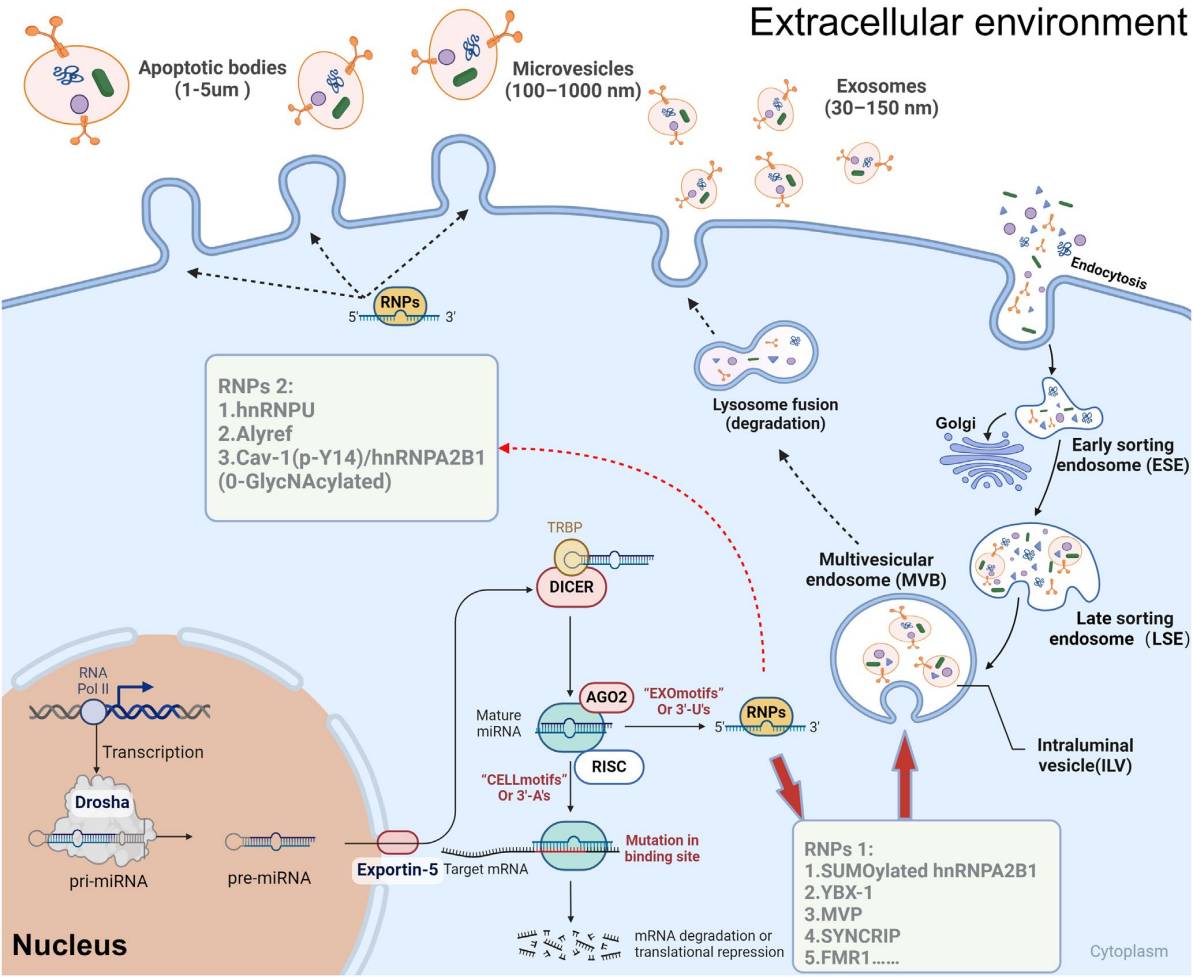
The biogenesis and release of EVs and miRNA sorting mechanism. The figure illustrates the biogenesis, sorting, and extracellular release of miRNAs via apoptotic bodies, microvesicles, and exosomes. The biogenesis of miRNAs begins in the nucleus, where primary miRNAs (pri‐miRNAs) are transcribed by RNA polymerase II and processed into precursor miRNAs (pre‐miRNAs) by Drosha. Pre‐miRNAs are exported to the cytoplasm via Exportin‐5 and further cleaved by Dicer into mature miRNAs. These mature miRNAs are then either retained in the cytoplasm for cellular functions or sorted into extracellular vesicles. miRNAs with CELL motifs or 3′‐adenylation are retained in the cytoplasm, where they bind to AGO proteins to form the RNA‐induced silencing complex (RISC), mediating mRNA degradation or translational repression. Conversely, miRNAs with EXO motifs or 3′‐uridylation are recognised by RNA‐binding proteins (RNPs), such as hnRNPA2B1, YBX1 and SYNCRIP, for selective sorting into exosomes or microvesicles. Group 1 RNPs (e.g. hnRNPA2B1, YBX1, MVP) facilitate miRNA incorporation into exosomes via multivesicular bodies (MVBs), while Group 2 RNPs (e.g., hnRNPU, Alyref, Cav‐1) direct miRNAs into microvesicles through plasma membrane budding. Exosomes (30–150 nm) are released into the extracellular environment via MVB fusion with the plasma membrane, while microvesicles (100–1000 nm) are shed directly from the plasma membrane. Additionally, apoptotic bodies (1–5 μm) formed during apoptosis carry miRNAs and other cellular contents. The secretion of miRNAs is also influenced by specific enzymes, such as nSMase2, which regulates exosome biogenesis. This comprehensive system ensures the selective sorting and release of miRNAs, enabling intercellular communication and gene regulation.

The potential of EVs in CVD is mainly achieved by nucleic acids, proteins, and lipid cargo they carry. In particular, miRNAs enriched in EVs emerged as important biomarkers of CVD [19]. Studies have shown that specific miRNAs show abnormal expression in CVD, such as AS and MI, while being able to mediate transcellular signal transmission through EVs [20, 21]. These miRNAs affect the development and progression of diseases by regulating gene expression in recipient cells and controlling biological processes such as cell proliferation, apoptosis, and migration [22]. We will detail the strong potential of miRNAs in Part 3. At the same time, the process of miRNA sorting into EVs is a highly specific mechanism involving the interaction of RNA‐binding proteins (e.g. Argonaute, HNRNPA2B1) with miRNAs, which are transported from the cytoplasm and encapsulated into EVs by recognising specific sequences, usually accompanied by the regulation of lipid microdomains and tetraspanin‐enriched regions (Figure 2).

The proteins carried by EVs, such as Alix, TSG101, HSP70, Syntenin‐1 and tetraspanins (e.g. CD63, CD81, etc.), not only reflect the characteristics of their source cells but also participate in the biogenesis and function of EVs. For example, HSP70 can promote the repair process after myocardial injury by playing a protective role in the stress response of cardiac cells [23]. In addition, Post‐Translational Modification (e.g. phosphorylation, glycosylation) of proteins in EVs is strongly associated with the progression of CVD, and these modifications may serve as potential biomarkers for early diagnosis and monitoring [24]. Finally, the lipid composition of EVs confers their stability and promotes cellular uptake. The membranes of EVs are rich in cholesterol, sphingomyelin, and phosphatidylserine, and these lipid components not only enhance the membrane stability of EVs but also help them target specific recipient cells in the cardiovascular system. Lipid components in EVs are essential for transcellular information transmission, especially in CVD, and can promote pathological changes and tissue repair by regulating cell–cell signal transmission [25].

### 2.3 EVs Isolation

The isolation of EVs presents a critical aspect of research in understanding their biological functions and potential applications. In the cardiovascular system, EVs are released by various cell types. Each cell type contributes uniquely to the pool of EVs, which carry specific molecular signatures reflective of their cellular origin and physiological state. Table 1 shows the specific markers for isolating and differentiating subpopulations of EVs from different cell types, including ECs, cardiomyocytes, fibroblasts and SMCs. Traditional differential ultracentrifugation remains a cornerstone technique for isolating EVs from cardiac cells and tissues due to its ability to process large sample volumes [26]. However, this method faces challenges such as low yield and co‐isolation of contaminants like protein aggregates and lipoproteins.

**TABLE 1:** Possible relevant EVs markers in cardiovascular research.

Cell type	Positive markers	Functions
Endothelial cells	CD31 (PECAM‐1), CD34, CD144	Angiogenesis, vascular homeostasis, inflammation
Cardiomyocytes	Cardiac troponin I, α‐actinin, yosin heavy chain	Intercellular communication during stress and repair cardiac
Fibroblasts	Vimentin, fibroblast activation protein (FAP), fibroblast‐specific Protein 1 (FSP1)	Extracellular matrix remodelling, fibrosis
Smooth muscle cells	Smooth muscle α‐Actin, SM22α	Vascular tone regulation, atherosclerosis
Platelets	CD41 (GPIIb), CD61 (GPIIIa), Platelet Factor 4 (PF4)	Promotion of thrombosis, involvement in inflammation
Monocytes/Macrophages	CD14, CD68	Mediation of inflammatory responses, plaque stability
Red blood cells	Glycophorin A, haemoglobin	Oxygen transport, modulation of vascular tone

*Note:* Possible relevant markers for EVs isolation and characterisation in cardiovascular diseases.

To address these challenges, size‐exclusion chromatography (SEC) [27] has been increasingly adopted in cardiovascular research. SEC effectively separates EVs from soluble proteins and lipoproteins, enhancing the purity of EV preparations obtained from plasma and serum samples. Affinity‐based isolation methods [28] have also gained traction, offering specificity by targeting EVs surface markers unique to certain cardiovascular cell types. For instance, immunoaffinity capture using antibodies against CD31 or CD144 can enrich endothelial cell‐derived EVs, while antibodies against cardiac troponin I can isolate EVs originating from cardiomyocytes. This targeted approach enhances purity and allows researchers to study cell type‐specific EVs functions and their contributions to CVD.

While significant progress has been made in EVs isolation techniques, achieving high yield, purity and specificity when isolating EVs from cardiovascular tissues or specific cell types remains challenging.

### 2.4 EVs Characterisation

Following isolation, the subsequent stage involves the comprehensive characterisation of EVs. According to MISEV2023 guidelines [29], EV characterisation should encompass multiple aspects: (1) physical properties: nanoparticle tracking analysis or dynamic light scattering, and assessing morphology and membrane integrity through high‐resolution imaging methods such as transmission electron microscopy or cryo‐electron microscopy (2) molecular markers: detecting the presence of EVs‐enriched proteins (positive markers) and the absence of non‐EVs contaminants (negative markers) using methods like Western blotting, flow cytometry or mass spectrometry. (3) functional assays: perform assays to demonstrate the functional capabilities of EVs, such as their effects on cell proliferation, migration or gene expression in recipient cells.

## 3 The Roles of EVs in CVD

**FIGURE 3:**
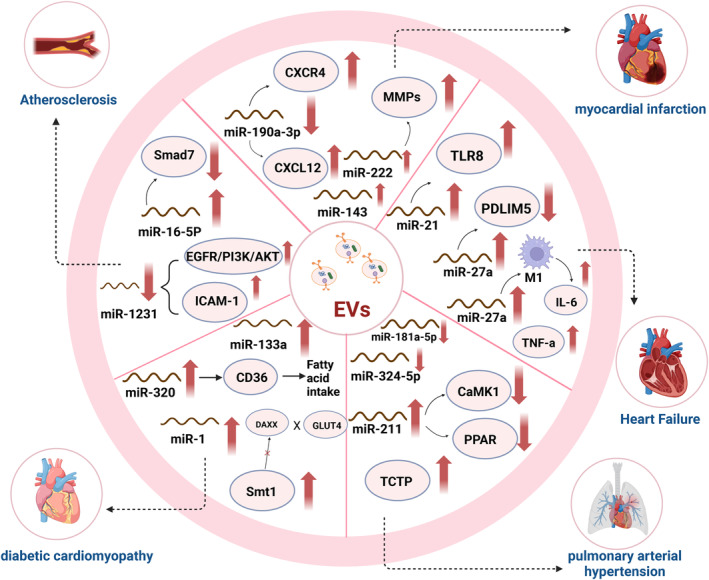
EVs in CVD. EVs originate from cardiovascular or non‐cardiovascular systems construct a complex modulating network for CVDs. The pathophysiological processes of CVDs are mainly influenced by diverse bioactive cargoes of EVs, such as nucleic acids, proteins and metabolites.

CVD encompasses a spectrum of diseases affecting the heart and blood vessels, including AS, MI, HF, pulmonary arterial hypertension (PAH), diabetic cardiomyopathy (DCM) and others. Subsequent sections will delve into the specific role of EVs in the development and progression of various CVD, providing a comprehensive understanding of their function in these diseases (Figure 3). We also summarise the critical role that miRNAs (Table 2).

**TABLE 2:** The EV‐miRNAs in the pathogenesis of CVD.

CVD phenotype	miRNA	Cell source	Target cell	Potential functional mechanism	Ref.
AS	miR‐185‐3p	mø	VSMCs	Targeting Smad7 to suppress TGF‐β/Smad signalling and promote vascular smooth muscle cell proliferation and migration	32
	miR‐16‐5p	mø	VSMCs	Targeting Smad7 to activate TGF‐β/Smad signalling and accelerate vascular smooth muscle cell proliferation and migration	33
	miR‐1231	monocytes	Coronary artery endothelial cells	Targeting miR‐1231 to release EGFR inhibition and enhance ICAM‐1/VCAM‐1 expression.	35
	miR‐4532	mø	EC	Targeting SP1 to activate NF‐κB P65 signalling and induce endothelial cell injury	37
	miR‐223	Blood cells	VSMCs	Targeting IGF‐1R to inhibit VSMC proliferation and migration	39
	miR‐19b‐3p	mø	VSMCs	Targeting JAZF1 to promote VSMC migration and proliferation	40
	miR‐503‐5p	mø	EC	Targeting CCNE1 to inhibit endothelial cell proliferation	41
MI	miR‐92a	CM	CF	Targeting Smad7 to promote myofibroblast activation	52
	miR‐1271‐5p	mø	CM	Argeting SOX6 to reduce cardiomyocyte apoptosis	53
	miR‐190a‐3p	Circulating exosomes	EPCs	Targeting CXCR4/CXCL12 to promote EPC proliferation and migration	51
HF	miR‐21	Mø Dendritic cells	CM mø	Targeting KBTBD7 to suppress excessive inflammation and cardiac dysfunction Targeting the vitamin D‐dependent antimicrobial pathway to modulate immune responses in leprosy	59,61
	miR‐27a	mø	CM	Targeting critical signalling pathways to promote cardiac hypertrophy	63
	miR‐378	CM	CF	Targeting fibrosis‐related genes to suppress myocardial fibrosis	64
	miR‐217	CM	CM	Targeting PTEN to promote cardiac hypertrophy and dysfunction	65
	miR‐192‐5p	Lipotoxic hepatocytes	mø	Targeting Rictor/Akt/FoxO1 signalling to activate macrophages	66
PAH	miR‐210	BMC	Pulmonary vascular endothelial cells	Targeting the iron–sulphur cluster assembly proteins ISCU1/2 to induce mitochondrial dysfunction and promote endothelial cell proliferation, contributing to pulmonary hypertension	73
	miR‐224‐5p and miR‐361–3p	PAECs	PAECs	Overexpression of SOX17 promotes the exosome‐mediated release of miR‐224‐5p and miR‐361–3p, which are internalised by injured PAECs in an autocrine manner, ultimately repressing the upregulation of NR4A3 and PCSK9 genes and improving endothelial function	74
DCM	miR‐320	CM	CM	Targeting the transcription of fatty acid metabolic genes, leading to cardiac lipotoxicity and dysfunction	77

Abbreviation: Atherosclerosis (AS), bone marrow‐derived cells (BMC), cardiac fibroblasts (CF), cardiomyocytes (CM), diabetic cardiomyopathy (DCM), endothelial cells (EC), endothelial progenitor cells (EPCs), heart failure (HF), macrophage (mø), myocardial infarction (MI), pulmonary arterial endothelial cells (PAECs), pulmonary arterial hypertension (PAH), small mother against decapentaplegic (Smad), vascular smooth muscle cells (VSMCs).

### 3.1 EVs In AS

AS is an inflammatory disease of large arteries [30] and is a leading cause of CVD and stroke. It is characterised by intimal plaque formation in large and medium‐sized arteries, from early to late atherosclerotic lesions containing MVs. Because of their effects on inflammation, thrombosis, neovascularization, cell survival and endothelial homeostasis, EVs may play a role in the development and progression of atherosclerotic lesions.

The development of AS begins with endothelial dysfunction, and studies have demonstrated that increased numbers of procoagulant circulating endothelial microparticles have been found in patients with acute myocardial ischemia, aggravating pre‐existing endothelial cell dysfunction. EVs have been shown to increase the expression of monocyte adhesion molecule receptors, promote monocyte adhesion to ECs, and mediate the development of microcalcification in atherosclerotic plaques [31]. Additionally, M1 macrophage‐derived EVs increase miR‐185‐3p targeting Smad7, promoting the pathological development of AS and altering blood lipid profiles while inhibiting cell proliferation and promoting apoptosis in vascular ECs of atherosclerotic mice [32]. Similarly, macrophage exosomal miR‐16‐5p aggravates AS progression by downregulating Smad7 expression [33]. At the same time, most in vitro studies have shown that MVs increase the release of proinflammatory cytokines, especially interleukin‐6 and interleukin‐8, from ECs and leukocytes, which in turn promote monocyte adhesion to the endothelium and facilitate monocyte migration to plaques. In addition to miRNAs, circularRNAs (circRNAs) in EVs can also regulate the pathology of levels of circNPHP4, which significantly increased in monocyte‐derived EVs from CVD patients [34]. CircNPHP4 translocates from monocytes into coronary endothelial cells (CAECs) in an EVS‐dependent manner and subsequently binds miR‐1231. Inhibition of miR‐1231 activates EGFR/PI3K/AKT and elevates intercellular adhesion molecule 1 (ICAM‐1) and vascular cell adhesion molecule 1 (VCAM‐1) expression on CAECs, thereby promoting monocyte adhesion to CAECs [35]. Taken together, these results suggest that microparticles released under pathological conditions can influence leukocyte and LDL infiltration in the subendothelial space, favouring early atherosclerotic lesion development. Meanwhile, aggregation of EVs can aggravate calcification formation and promote vasoactive responses.

EVs derived from various cells are important regulators of the pathological atherosclerotic process during the progressive stages of the disease. For example, EVs derived from CD4 (+) T lymphocytes can stimulate cholesterol accumulation in macrophages in vitro and mediate the synthesis of pro‐inflammatory cytokine TNF‐α by macrophages through phosphatidylserine receptors [36]. Macrophage‐derived exosomes miR‐4532 significantly disrupt human umbilical vein ECs function by targeting SP1 and downstream NF‐κB p65 activation, with increased expression of ET‐1, ICAM‐1 and VCAM‐1 in ECs, along with decreased endothelial nitric oxide synthase (eNOS) expression in ECs, exacerbating foam cell formation and exosomes miR‐4532 transport to ECs [37].

Vascular smooth muscle cells (VSMCs) play an essential role in atherosclerotic lesion development [38]. EVs of various contents can also control VSMC proliferation and migration. MVs containing miR‐223 can penetrate the vessel wall, inhibit vascular smooth muscle cell proliferation and migration, and reduce plaque size [39]. MiR‐19b‐3p also promotes the proliferation and migration of VSMC by inhibiting JAZF1 levels in VSMCs, thereby promoting the development of AS [40]. MiR‐503‐5p inhibits the proliferation and angiogenic effects of ECs by simultaneous transfer of EVs into ECs and VSMCs while promoting the proliferation and migration of VSMCs by down‐regulating the expression of smad family members 1, 2 and 7 small molecules [41].

### 3.2 EVs In MI

Atherosclerotic plaque rupture and subsequent haemorrhage lead to acute myocardial infarction (AMI). It is characterised by coronary artery occlusion as well as massive cardiomyocyte death [42]. Many studies have revealed the regulatory role of EVs and their cargo in MI.

In AMI, cardiac myocytes increase the secretion of EVs containing cardiac‐specific non‐coding RNAs and significantly protect the heart. For example, miRNA‐133 has anti‐fibrotic effects, miR‐1 has specific antioxidant effects, and miRNA‐499 has anti‐apoptotic properties [43]. Exosomes secreted by cardiac progenitor cells under hypoxic conditions improved cardiac function and reduced fibrosis [44]. It has also been noted that some AMI patients or ischemic heart disease patients have impaired cardioprotection of circulating EVs because they contain cargo such as miRNAs miR‐193a‐3p [45], miR‐1915‐3p [46], miR‐204 [47] and circRNAs, lncRNAs [48] that are reduced. In AMI conditions, these EVs are secreted into the circulation by various donor cells and subsequently internalised by different recipient cells, activating different signalling pathways and ultimately regulating the pathology of AMI [49, 50]. Different cell‐derived EVs are also important regulators of MI. MiR‐190a‐3p was significantly down‐regulated in exosomes from cardiomyocyte supernatant under hypoxic environment, and these exosomes could upregulate CXCR4 and CXCL12 expression in endothelial progenitor cells; in vitro, functional assays showed that miR‐190a‐3p overexpression inhibited cell viability, proliferation, migration, adhesion and tube formation of endothelial progenitor cells (EPCs); while miR‐190a‐3p inhibitor had the opposite effect, so decreased miR‐190a‐3p could promote proliferation, migration, adhesion and tube formation of endothelial progenitor cells, so down‐regulation of miR‐190a‐3p in circulating exosomes may have a protective effect on myocardial injury [51]. MiR‐222 and miR‐143, the relatively most abundant exosomes secreted by cardiomyocytes under ischemic conditions, showed higher levels of metalloproteinases (MMPs), stimulating the formation of capillary‐like structures and promoting cardiac angiogenesis. MiR‐92a was significantly upregulated in cardiomyocyte‐derived exosomes and fibroblasts isolated after MI promoting myofibroblast activation [52]. In contrast, overexpression of AK139128 by exosomes derived from hypoxic cardiomyocytes stimulated apoptosis and inhibited proliferation, migration, and invasion in cardiac fibroblast. MiR‐1271‐5p attenuates myocardial injury in AMI by decreasing hypoxia‐induced cardiomyocyte apoptosis through downregulation of SOX6 expression [53]. However, M1‐Exos has anti‐angiogenic effects and accelerates MI injury. They also exhibited highly expressed pro‐inflammatory miRNAs, such as miR‐155 [54].

### 3.3 EVs in HF

Pathological cardiac hypertrophy results in enlarged cardiomyocytes, fibrosis in the tissue between cells, and inadequate blood supply. These factors contribute to cardiac dilation and dysfunction in both systolic and diastolic phases, ultimately leading to HF [55].

In recent years, numerous investigations have underscored the involvement of EVs and EVs‐derived miRNAs and proteins, originating from various cellular sources, in the pathological mechanisms underlying chronic heart failure (CHF) [7]. For example, decreased levels of EVs have been observed in patients with acute heart failure after cardiac surgery with cardiopulmonary bypass [56]. In contrast, a significant increase in EVs released from cardiomyocytes was found in a CHF rat model. Additionally, under angiotensin II stimulation, the number of EVs released from white adipose tissue of the accessory testis was significantly increased [57].

Compelling evidence indicates that in pathological states, EVs derived from cardiac fibroblasts transport miRNAs into cardiomyocytes, actively inducing hypertrophic growth. For example, in HF patients following AMI, levels of miR‐192, −194 and −34a are markedly elevated in circulating exosomes; the levels of miR‐194 and miR‐34a in circulation were also found to have a positive correlation with the left ventricular diastolic dimension and ejection fraction in patients with HF induced by AMI [58]. MiR‐21 can target KBTBD7 and attenuate maladaptive inflammatory responses, primarily by promoting the pro‐inflammatory response triggered by damage‐associated molecular patterns in macrophages via the p38 and NF‐κB pathways. Hence, it may serve as a potential therapeutic target [59]. Additionally, miR‐21 has been shown to enhance the expression of Toll‐like receptor (TLR) 8 in macrophages, thereby inducing the production of pro‐inflammatory cytokines [60]. This miRNA interacts with various genes, inhibiting their functions and supporting antimicrobial actions [61]. MiR‐21‐5p EVs support cardiac repair through survival mechanisms, while cardiac fibroblast EVs stimulate hypertrophic signalling pathways [62]. Fibroblast‐derived exosomes enriched with miR‐27a are transported into cardiomyocytes, where they inhibit PDLIM5 expression and exacerbate cardiac hypertrophy in MI‐induced CHF models, a process closely linked to oxidative stress, which significantly contributes to cardiomyocyte dysfunction and myocardial hypertrophy in CHF [63]. Decreased miR‐30d levels have been observed in circulating EVs isolated from ischemic heart failure rodents (rats and mice) and patients; miR‐30d is mainly released by cardiomyocytes in EVs and mediates paracrine signalling in cardiac fibroblasts, leading to fibroblast activation and proliferation by inhibiting integrin α5 expression. Excessive myocardial fibrosis is a primary pathological process in cardiac remodelling and the development of HF; studies [64] have shown that miR‐378 has a vital role in the regulation of cardiac fibrosis, and miR‐378 is secreted by cardiomyocytes after mechanical stress and inhibits p38 MAP kinase phosphorylation to MKK6 in cardiac fibroblasts through a paracrine mechanism. In contrast [65], overexpression of miR‐217 in CHF patients has been found to aggravate pressure overload‐induced cardiac hypertrophy, fibrosis, and cardiac dysfunction. MiR‐192‐5p overexpression induced M1 macrophage activation and increased expression of inducible nitric oxide synthase, interleukin‐6 and tumour necrosis factor alpha [66]. Elevated miR‐192 expression has also been associated with hypertrophic cardiomyopathy and circulating minute RNA may be a novel marker representing CVD [67]. Notably, Overexpression of miRNA‐132 could protect against apoptosis and oxidative stress in HF [67].

### 3.4 EVs in PAH

PAH refers to the clinical and pathophysiological syndrome caused by changes in pulmonary vascular structure or function due to various heterogeneous diseases (etiologies) and different pathogenesis. These changes cause increased pulmonary vascular resistance and pulmonary arterial pressure, which in turn lead to right heart failure or death.

Circulating EVs are elevated in PAH patients, and their composition is also altered [68]. Studies have shown that platelet‐derived EVs from PAH patients evade lysosomes following internalisation in human pulmonary artery endothelial cells (hPAEC) and may be involved via the P‐selectin glycoprotein ligand 1 pathway, thereby inducing hPAEC activation and angiogenesis in vitro [69]. Additionally, monocytes overexpressing HERV‐K dUTPase release EVs containing HERV‐K dUTPase, which led to pulmonary hypertension in mice and induce associated endothelial‐mesenchymal transition and pro‐inflammatory molecules interleukin‐6 as well as VCAM‐1 [70]. Increased translation‐controlled tumour protein (TCTP) expression was found in patients with heritable PAH. Co‐culture assays showed that exosomes transferred TCTP from EC to pulmonary artery SMCs, allowing endothelium‐derived TCTP to confer proliferation and apoptosis resistance, promoting PAH development and progression.

Various EVs containing different miRNA cargoes play an important role in the regulation of PAH development as well as cell‐to‐cell crosstalk. In preclinical and idiopathic PAH studies, p.KLF2‐induced exosomal miR‐181a‐5p and miR‐324‐5p are reduced in heritable PAH with the H288Y KLF2 mutation, leading to increased target gene expression and attenuated pulmonary vascular remodelling via Notch4, ETS1, and other vascular homeostasis regulators [71]. In pulmonary hypertension (PH) rat model, plasma exosomes concentration increased, and miR‐211 in exosomes was upregulated, while the expression of CaMK1 and PPAR‐γ decreased in lung tissue, promoting PH and pulmonary arterial smooth muscle cell proliferation in rats [72]. Endogenous bloodborne delivery of miR‐210 to pulmonary vascular endothelial cells also promotes PH in a mouse model [73]. In PH patients and SU5416/hypoxia‐induced PH mice, SOX17 expression was down‐regulated in dysfunctional HPAEC remodelling pulmonary artery endothelial cells. Overexpression of SOX17 promoted exosomes‐mediated miR‐224‐5p and miR‐361–3p release, ultimately inhibiting the up‐regulation of NR4A3 and PCSK9 genes and improving endothelial function [74].

### 3.5 EVs In DCM

DCM is a progressive heart disease that occurs in diabetic patients and can independently cause myocardial damage and reduced cardiac function. It can develop into HF over time, and its pathogenesis involves multiple aspects, such as oxidative stress [75].

The role of EVs in diabetes‐related cardiovascular complications has been extensively discussed and explored by various laboratories, demonstrating a close association between DCM and EVs [76]. Myocardial steatosis is a hallmark of DCM, and miR‐133a levels are elevated in patients with Type 2 diabetes compared with healthy subjects. Circulating miR‐1 and miR‐133a levels are significantly elevated in mice fed with high‐fat diet compared with control animals, correlating a higher myocardial steatosis. Levels of miR‐1 and miR‐133a were also higher in exosomes released from HL‐1 cardiomyocytes similarly loaded with lipids. Research has shown that miR‐320 exacerbated the induction of DCM, and its expression by targeting CD36 (fatty acid translocase) resulted in increased fatty acid uptake, leading to lipotoxicity in the heart [77]. Cardiac EC‐derived exosomes can also be transferred to cardiomyocytes increasing Mst1 protein content, which disrupts Type 4 glucose transporter (GLUT4) membrane translocation by reducing the interaction between Daxx and GLUT4 and enhancing the association between Mst1 and Daxx, thereby inhibiting glucose uptake of under diabetic conditions [78]. However, other cell‐derived EVs may be involved in DCM, and more studies are needed relative to these important issues.

## 4 Targeting EVs for Diagnosis and Treatment of CVD

### 4.1 Diagnosis Potential of EVs in CVD

CVD is the leading cause of death worldwide, so early and rapid diagnosis of CVD is essential to improve treatment outcomes and save patients' lives [79]. Recent studies have demonstrated that EVs are powerful potential candidates for diagnostic biomarkers of CVD [80]. EVs have unique advantages: first, they are easily accessible and analysed [80], and second, the biogenesis of EVs is associated with cellular processes in disease, and their complex components, combined, may better reflect disease pathology than any single molecular marker. Significantly elevated levels of miR‐27a, miR‐28a and miR‐34a in circulating EVs have been observed in patients and chronic heart failure (CHF) rats [7]. An increase in miR‐92b‐5p levels in the exosomal miR‐92b subgroup of diabetic cardiomyopathy (DCM) patients has been reported [81], while in AMI patients, circulating miR‐1, 208 and 499 levels were significantly increased within 1–24 h, and peaked at 3–6 h after AMI [82]. The protein cargo of EVs can also be used as biomarkers to assess cardiovascular risk or pathology [58]. In addition, bilayer membranes rich in cholesterol and sphingolipids of EVs provide strong protection and preservation of cargoes therein. For example, the phospholipid bilayer of exosomes contains abundant lipids, such as ceramide, sphingomyelin and phosphatidylcholine, protecting various bioactive molecules from enzymatic hydrolysis and the external environment.

In addition to diagnosis, EVs are associated with the prognosis of certain CVD and predict the progression of CVD. In patients who experienced new‐onset heart failure within 1 year after AMI, increased levels of three p53‐responsive miRNAs (miR‐192, miR‐194, miR‐34a) were detected early in the recovery phase, which could predict the risk of ischemic heart failure after AMI [58]. An analysis of 10 miRNAs involved in vascular performance regulation in plasma and circulating microvesicles from 181 patients with stable coronary artery disease demonstrated that microvesicles containing miR‐126 and miR‐199a but not free circulating miRNA expression predicted the occurrence of cardiovascular events in these patients [83]. Furthermore, endothelial dysfunction assessed by plasma endothelial microparticle levels could independently predict future cardiovascular events in patients at high risk for coronary heart disease [84]. In summary, EVs hold promise as biomarkers of CVD.

### 4.2 Therapeutic Potential of EVs in CVD

EVs have many unique characteristics, such as ease of acquisition, modification, low immunity and ease of crossing the cellular barrier, which makes them ideal for cell‐free therapeutics or drug carriers.

Based on available information, EVs from various sources have been extensively evaluated in animal models for the treatment of ischemic heart disease. Thereby EVs can be used as a promising cell‐free therapeutic strategy for CVD treatment. Mesenchymal stem cell (MSC)‐derived EVs are currently the most studied EVs in CVD treatment, MSCs derived exosomes miR‐143‐3p regulate autophagy through the CHK2‐Beclin2 pathway and effectively reduce apoptosis [85] and in neonatal mouse cardiomyocytes cultured by hypoxia and serum deprivation, MSC secreted exosomes miR‐125b reduces autophagic flux and cell death [86], in addition to reducing autophagy, apoptosis, and proptosis, cardiac MSCs delivered exosomes after MI enhance cardiac angiogenesis, promote cardiomyocyte proliferation, and protect cardiac function [87]. In addition to MSC‐derived EVs, EVs released from other cells can also improve the development and progression of CVD, such as EVs from cardiomyocytes, ECs, platelets, endothelial colony‐forming cells and cardio‐sphere‐derived cells. For example, the synthesis and secretion of exosomes from cardiomyocytes are increased under glucose‐deficient conditions, and they are internalised by ECs to increase glucose transport [88]. Hypoxia‐induced circWhsc1 in EC‐derived EVs induced cardiomyocyte proliferation after MI in adult mice, reduced cardiac fibrosis, and restored cardiac function [89], and endothelial colony‐forming cell‐derived exosomes were also found to rescue autophagic flux and inhibit SIAP1L2 expression by releasing miR‐21‐5p, thereby preventing AS induced vascular injury [90].

In recent years, the combination of EVs with biomaterials has improved the therapeutic potential of EVs to a greater extent. For instance, combining injectable heat‐responsive hydrogels with EVs enhances the stability of EVs in vivo and allows their release at different temperatures, which significantly improves neovascularization after severe limb ischemia, reduces muscle injury, and restores limb function [91]. Additionally, loading a NOD‐like receptor 3 inflammasome inhibitor into platelet‐derived EVs is expected to be used for targeted drug delivery for the treatment of AS [92]. By embedding MSC‐derived exosomes in hyaluronic acid hydrogels, an injectable ExoGel was created and tested. Injecting ExoGel into the pericardial cavity of rats with heart failure induced by transverse aortic constriction reduced left ventricular chamber size and preserved wall thickness, and further studies verified the feasibility and safety of ExoGel injection in a porcine model [93].

### 4.3 Enhancing EVs Therapeutics

Although EVs hold great promise as non‐cellular therapeutic tools, challenges remain, including low yield, inability to isolate specific EV components and non‐specific delivery. Enhancing the therapeutic efficacy of stem cell‐derived EVs requires addressing these limitations. Recent advances in biomedical engineering, particularly in hydrogel applications, have significantly improved EVs delivery [94]. For example, EPC‐derived EVs delivered via injectable hydrogels have been shown to promote angiogenesis and improve myocardial hemodynamics in a rat model of myocardial ischemia [95]. Additionally, a conductive hydrogel synergistically combines the cardiac regenerative capabilities of exo with the conductive properties of poly‐pyrrole‐chitosan to improve cardiac functioning via promoting angiogenesis and inhibiting apoptosis, as well as resynchronizing electrical conduction [96].

Genetic engineering techniques, including gene editing, knockout, knock‐in, transgenics and gene silencing, further expand EVs therapeutic applications. For example, the gene editing technology CRISPR‐Cas9 has been used to treat genetic diseases, such as sickle cell anaemia and muscular dystrophy. Numerous studies have shown that genetic engineering can modify EVs and reduce off‐target effects, thereby improving therapeutic efficacy [97]. For instance, combining bone marrow‐derived mesenchymal stem cell‐derived exosomes with ischemic myocardial targeting peptide under hypoxia via bioorthogonal chemistry, followed by intravenous injection, demonstrated specific targeting of ischemic lesions in the injured heart and exerted significant cardioprotective functions after MI [98]; conjugating exosomes with cardiac homing peptide using a targeting peptide has been shown to improve exosome survival [99]. Furthermore, advancements have been made to improve the uptake and release of exosomes from cells. Initially, a pH‐sensitive fusion peptide was used to achieve the fusion of endosomal and exosomal membranes within cells, enhancing exosome uptake and release. Subsequently, the cell‐penetrating peptide stearylated octa arginine was utilised to induce large protein endocytosis by simple modification of the exosomal membrane, significantly improving the uptake efficiency of cellular EVs [100].

## 5 Conclusion

Over the past decades, EVs‐based therapies have undergone revolutionary advances, particularly in understanding EV biology, their vital role in CVD physiology and pathology, and their clinical potential in diagnosis and treatment. However, further studies are needed to better understand the specific cargo sorting, internalisation processes, and cargo release of EVs in cardiovascular contexts, which will help resolve functional inconsistencies of similar EVs reported in different studies. This remains an evolving field, and future mechanistic studies will shed light on determinants of EV function. Variations in EVs components under different pathophysiological conditions suggest that EVs have the potential to distinguish specific subtypes or stages of CVD. Therefore, evaluating the use of EVs for a more precise classification of CVD status is warranted. While the therapeutic benefits of EVs for ischemic heart disease have been demonstrated in large animal models, their application in CVD treatment is still in its infancy. More exploration is needed to maximise EVs loading efficiency, enhance target selectivity, and combine EVs with classical therapeutic biomaterials.

## Author Contributions

**Hanbin Li:** conceptualization (equal), data curation (equal), visualization (equal), writing – original draft (equal), writing – review and editing (equal). **Lu Wang:** conceptualization (equal), data curation (equal), visualization (equal), writing – original draft (equal), writing – review and editing (equal). **Hongxin Cheng:** data curation (supporting), visualization (supporting). **Qing Zhang:** data curation (supporting), visualization (supporting). **Shiqi Wang:** data curation (supporting), visualization (equal). **Wen Zhong:** data curation (supporting), visualization (supporting). **Chengqi He:** supervision (lead), writing – review and editing (lead). **Quan Wei:** conceptualization (lead), funding acquisition (lead), supervision (lead), writing – original draft (equal), writing – review and editing (equal).

## Conflicts of Interest

The authors declare no conflicts of interest.

## Data Availability

Data sharing not applicable to this article as no datasets were generated or analysed during the current study.
